# Large sample size and nonlinear sparse models outline epistatic effects in inflammatory bowel disease

**DOI:** 10.1186/s13059-023-03064-y

**Published:** 2023-10-05

**Authors:** Nora Verplaetse, Antoine Passemiers, Adam Arany, Yves Moreau, Daniele Raimondi

**Affiliations:** Department of of Electrical Engineering, Katholieke Universiteit Leuven, Leuven, Belgium

**Keywords:** Genome interpretation, Machine learning, Neural networks

## Abstract

**Background:**

Despite clear evidence of nonlinear interactions in the molecular architecture of polygenic diseases, linear models have so far appeared optimal in genotype-to-phenotype modeling. A key bottleneck for such modeling is that genetic data intrinsically suffers from *underdetermination* ($p \gg n$). Millions of variants are present in each individual while the collection of large, homogeneous cohorts is hindered by phenotype incidence, sequencing cost, and batch effects.

**Results:**

We demonstrate that when we provide enough training data and control the complexity of nonlinear models, a neural network outperforms additive approaches in whole exome sequencing-based inflammatory bowel disease case–control prediction. To do so, we propose a biologically meaningful sparsified neural network architecture, providing empirical evidence for positive and negative epistatic effects present in the inflammatory bowel disease pathogenesis.

**Conclusions:**

In this paper, we show that underdetermination is likely a major driver for the apparent optimality of additive modeling in clinical genetics today.

**Supplementary information:**

The online version contains supplementary material available at 10.1186/s13059-023-03064-y.

## Background

Since the sequencing of the first whole human genome in 2003 and the advent of high-throughput sequencing techniques [1], clinical and population genetics have been blooming fields of research. Every year, an increasing number of genetic studies are published worldwide, dedicated to understanding the relationship between genotype and phenotype, which is a crucial step towards precision medicine and other health applications [2, 3]. Nevertheless, given that each individual genome contains about three million variants, together with the challenges in gathering large, homogeneous cohorts because of limited phenotype incidence, sequencing cost, and batch effects, most of these studies suffer from a limited sample size *n* relative to the number of variants *p* (also called the $p \gg n$ setting in statistics). This *underdetermination* of genetic datasets has indeed been a crucial problem so far, limiting the statistical power of analysis approaches and contributing to puzzling results, such as the missing heritability problem [4, 5]. For this reason, the go-to methods for genomic data analysis have historically always been additive (linear) models, such as univariate analysis of association (i.e., genome-wide association studies (GWAS) [6]), polygenic risk scores (PRS) [7], and linear mixed models [8, 9].

Relying on additive models has until now appeared to be optimal in the genetic context [10], thanks to their interpretability and simplicity, which conveniently translates into robustness at unfavorable *n*/*p* ratios. Researchers thus adopted this *lens* of additivity in their investigations to address the ubiquitous underdetermination of their datasets, notwithstanding biological arguments for the existence of nonlinear genetics effects, such as epistasis [5, 11–17]. With the blooming of complex machine learning (ML) methods (e.g., deep learning) in the life sciences [18–20] and the growth of the available sample sizes because of advances in sequencing technologies [1] and the accompanying decrease in cost, it is now timely to consider whether more sophisticated approaches could also benefit genetic data. Such approaches could allow us to perceive aspects of the genetics landscape that are currently masked by the omnipresent lens of additivity.

Early attempts to apply neural networks (NN) to genome interpretation (GI), namely the explicit modeling of the relationship between genotype and phenotype, did not succeed in outperforming additive models [10, 21]. The debate on the opportunity of using nonlinear models is still widely open in clinical genetics [10, 21–29] and agricultural biotechnology [30–34]. However, the current effectiveness of additive modeling in genetics raises more questions than answers [15]. Most of the molecular mechanisms producing phenotypes described by systems and molecular biology studies are replete with nonlinear interactions between the components of extremely complex systems [5, 11–17, 35]. In these disciplines, pure additive effects are as uncommon as they are widespread in current genetics literature.

From a data science perspective, we see two main limitations that could have prevented NN models for GI from outperforming additive approaches. First, most of the NNs applied on human genetic datasets of large sample sizes used SNP array data that has been prefiltered with a GWAS-based univariate variant selection [21, 26–29], thereby possibly removing a priori much of the nonadditive interaction signal from the input. Second, NN GI attempts on WES/WGS data have been only sporadic and mostly restricted to very small sample sizes [22–24, 36, 37], while it is empirically clear that deep learning methods require large datasets to perform best. If nonlinear models can outperform linear ones, a key question is then how large a dataset needs to be before a nonlinear model starts offering any advantage.

In this article, we test the hypothesis that underdetermination is one of the major drivers for the apparent optimality of additive models in genetics. To do so, we exploit the intrinsic sparsity of biological networks to build the smallest NN model possible that is still capable of nonlinear inference and apply it to one of the largest available WES case–control datasets. This is a dataset for inflammatory bowel disorder (IBD), which constitutes an ideal test case for our study given its polygenic nature and high heritability estimates.

We show that once a sufficiently large sample size is reached, NNs reliably outperform conventional additive approaches. Moreover, we show (1) how this result provides empirical evidence that, given enough samples and a model able to detect them, epistatic effects start to emerge from the data and (2) that positive/negative epistasis plays a role in the genetic mechanism underlying IBD. Our study indicates that the main reason for the effectiveness of additive models for the analysis of genetic data is their robustness at small sample sizes, thereby recontextualizing them as a situational and temporary necessary solution instead of the undisputed *statistical model* for genetics. Our results indicate that as larger genetic datasets become available, we can envision a systematic nonlinear advantage for NN models applied to GI.

## Results and discussion

### Small, biologically sparsified NNs outperform additive and nonlinear baselines

To address the predominant underdetermination of genetic datasets, but still benefit from nonlinear modeling, we minimized the gap between the number of samples and the number of model parameters by applying the smallest possible NN (but which could still perform nonlinear inference) to one of the largest whole exome sequencing (WES) inflammatory bowel disease (IBD) cohorts available.

**Fig. 1:**
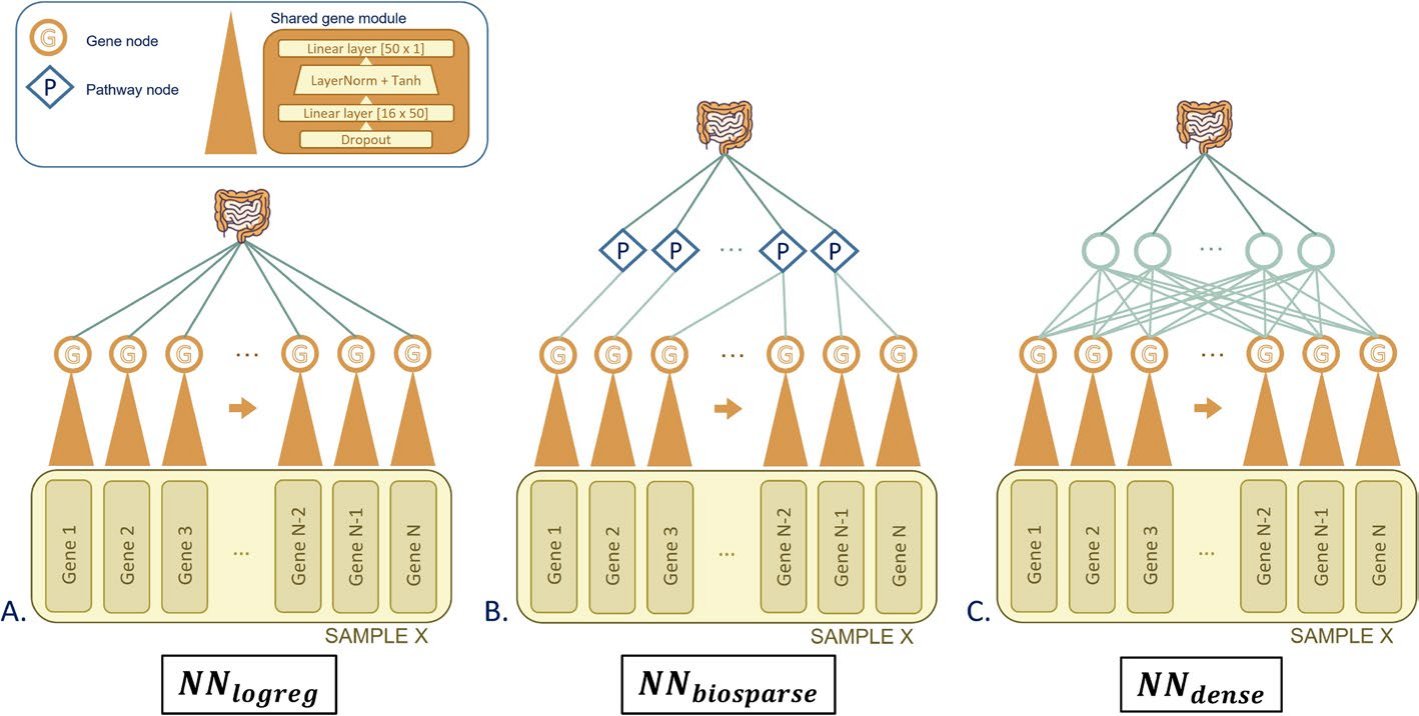
Panel overview of NN architectures, each build on top of the shared gene module with increasing complexity. Panel **A** shows NN_logreg_, the simplest architecture connecting the gene neurons *G* directly to the output, implementing a LogReg of the |*G*| neurons. Panel **B** illustrates NN_biosparse_ with connections between gene neurons *G* and pathway neurons *P* based on the KEGG database. The model in panel **C**, NN_dense_, adds a fully connected hidden layer to increase model expressiveness

Similarly to [36, 37], to obtain a maximal reduction of the number of parameters in our NN, we start by using a compact, gene-centric representation of the input WES data in which each gene is represented by the observed *mutational load* it carries (see the “Material and methods” section). Genes are thus the base biological semantic entity in our models (see input data in Fig. 1). Several variations of this encoding have been explored as well (see Additional file 1: Fig. S1, Additional file 2: Table S1, Additional file 3: Table S2) without observing performance improvement.

In Table 1, we benchmark three NN architectures specifically designed for the WES-based case–control IBD prediction against conventional methods, such as an additive model and RF. We tested several alternatives for the additive model. In Table 1, we only show the best performing additive approach, which is a logistic regression with $L_2$ regularization. We could not add polygenic risk scores (PRS) to this benchmark, because most of the variants found by GWAS are noncoding, resulting in only 42% of IBD GWAS variants [38, 39] present in our WES IBD dataset. A PRS computed on these 42% of GWAS variants produces a test ROC AUC of 0.563. Methodological details and additional analyses on this variant selection can be found in Additional file 4: Note S1.

**Table 1:** Benchmark of our NN architectures specifically designed for the WES-based case–control IBD prediction against conventional methods

Model	ROC AUC^a^	Number of parameters
Best additive model	0.728 (0.00599)	1,734,301
Random forest	0.688 (0.00578)	
NN_logreg_	0.753 (0.0117)	24,379
NN_biosparse_	0.758 (0.00689)	25,503
NN_dense_	0.743 (0.00944)	6,515,063
NN_linear_	0.717 (0.0261)	25,503

^a^Performance given as mean (standard deviation) of test set ROC AUC from 10 different full threefold cross-validation runs with identical splits across models

The architectures of the NNs we tested (as reported in Table 1) are shown in Fig. 1 by increasing complexity from left to right. In all of them, a shared gene module processes separately the features describing each gene (see the “Material and methods” section) and the differences between the architectures lie in the arrangement of the modules connecting the $|G| =$ neurons and the output neuron. These NN architectures were designed so that they represent three levels of expressivity (i.e., complexity of the decision boundaries they can learn). This translates in their increasing capability of modeling certain categories of interactions between the |*G*| neurons. In the simplest model, called NN_logreg_ (see Fig. 1A), each gene latent representation produced by the shared gene module is connected to the output neuron similarly to a logistic regression (LogReg). This model will therefore not be able to model any interactions between the |*G*| neurons. The second model (see Fig. 1B) represents an intermediate level of expressivity whereby two gene neurons are connected to and can thus interact in at most one hidden neuron of the next layer. The choice of the connection arrangements was based on the Kyoto Encyclopedia of Genes and Genomes (KEGG) database [40] with the hidden nodes representing the KEGG pathways and the connection for each gene being picked out of the known KEGG gene-pathway relations. Since each neuron and each connection in this architecture has a biological meaning, we refer to this model as a biologically sparsified NN (NN_biosparse_). Finally, Fig. 1C shows an NN with a standard densely connected hidden layer between gene neurons and output (NN_dense_). This way all gene neurons are connected to all hidden neurons, making it possible to model any kind of interaction between them (universal approximator) [41].

In Table 1, we show the ROC AUC obtained by each model on IBD case–control discrimination, alongside their number of trainable parameters. Our NN models outperform the best performing RF (two-sided *t*-test, corrected for correlation between ROC AUC measurements [42]: *p*-value $p=5.77\textrm{e}{-7}$) and the best additive model (corrected two-sided *t*-test [42] $p=0.00457$). Among the three NN architectures we presented, NN_biosparse_, which has the lowest number of parameters, is also the best performing model. A detailed table of RF performance on different data representations can be found in Additional file 5: Table S3.

### Sample size can explain the apparent optimality of additive models in genetics

**Fig. 2:**
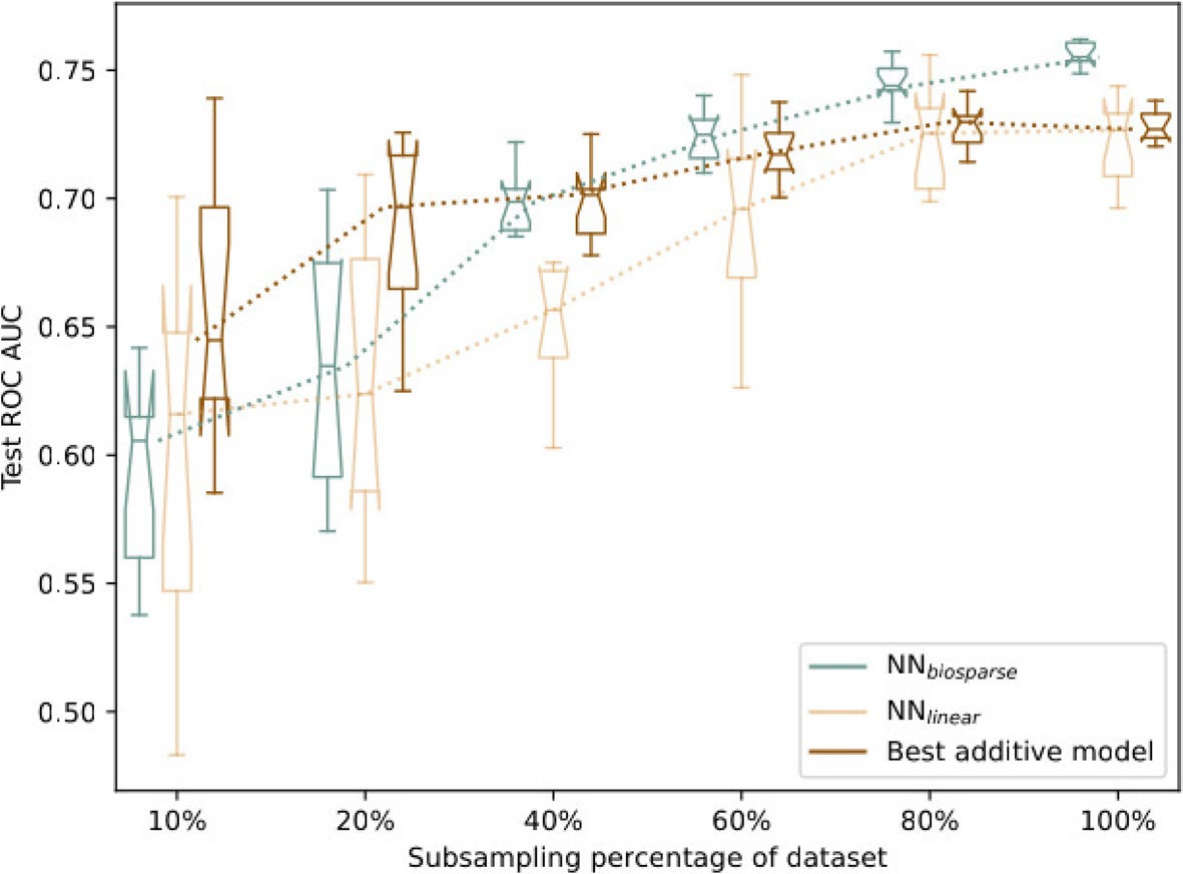
Performance using different random subsets containing 10%, 20%, 40%, 60%, 80%, and 100% of the dataset. Models shown are the best additive model ($\text {L}_2$ penalty), NN_biosparse_ and NN_linear_. Performances are measured using ten different threefold cross-validation runs, using identical splits for the three models

Table 1 shows that our NN models outperform the best results obtained by classical additive approaches, which is not always the case in genetic studies [10, 21, 22, 28, 30–33]. To investigate in more details at which conditions this advantage appears, we show in Fig. 2 the comparison of the prediction performances of these two approaches in function of the sample size available during training. By looking at the trajectories of these models, we can see that when at least 80% (3038) of samples are used for training, our best approach (NN_biosparse_) starts to outperform the additive model and the improvement eventually becomes statistically significant. This empirically demonstrates that for this dataset the optimality of additive models is not general, but sample size-dependent.

The balance between model expressivity (ability to learn nonlinear patterns) and prediction quality is nevertheless quite delicate in this ($p \gg n$) dataset, and is directly related to the bias–variance trade-off [43, 44]. Bias and variance can be considered as two competing qualities of statistical models. Bias refers to the error the model makes because of incorrect assumptions and limited expressive power, and it is linked to *underfitting*. Variance represents the ability of the model to learn small variations (and noise) in the training data, and relates to the risk of *overfitting*. Simple additive approaches have high bias but, while their ability to model complex patterns is limited, the uncertainty about optimal model parameters will decrease faster than for more complex models. Genetic datasets have thousands or even millions of possibly noisy observations (variants) for each sample, and the intrinsic simplicity of additive model helps them handle the resulting uncertainty. At small sample size, additive models can learn only a simple hypothesis from the data, while more expressive (high variance) models such as NNs are at greater risk of achieving poor generalization.

#### Absence of evidence is not evidence of absence

Figure 2 empirically shows that even if at low sample sizes additive models are optimal, there can exist a sample size threshold *t* above which certain nonlinear patterns become detectable by sufficiently expressive models. In the case of this IBD cohort and our NN_biosparse_, we were able to empirically identify $t \approx 3000$, since it happens to be lower than the dataset sample size ($t < n$). We can speculate that the same behavior could be observed in several other real life genetic datasets for appropriately large sample sizes. Therefore, to have a chance to be truly conclusive and comprehensive, the currently ongoing debate on the use of additive versus nonlinear models on genetic datasets should make sure to investigate the behavior of both models across a sufficiently wide range of sample sizes. This is crucial to ensure that the apparent optimality of additive models does not come from the fact that $t>n$ in the datasets under consideration. Furthermore, this might help to get a more complete molecular picture, thereby addressing the well-known missing heritability problem.

To exclude that the behavior in Fig. 2 is simply the result of the different format of the input representations between the additive model (which takes as input a vector of all the variants) and our NNs (which use our gene-centric data representation, see the “Material and methods” section), we ran an additional experiment. We add a third model to Fig. 2 to compare our NN_biosparse_ with an NN with the same architecture, but with identity functions instead of the hyperbolic tangent $\tanh$ nonlinear activations (NN_linear_). This makes this NN_linear_ effectively equivalent to a linear model. Again, we see the same pattern as for the best additive model, indicating that the higher performances at high sample size emerge from the nonlinearity of the model and not from the different input encoding per se.

### Empirical evidence for positive/negative epistasis in IBD

**Fig. 3:**
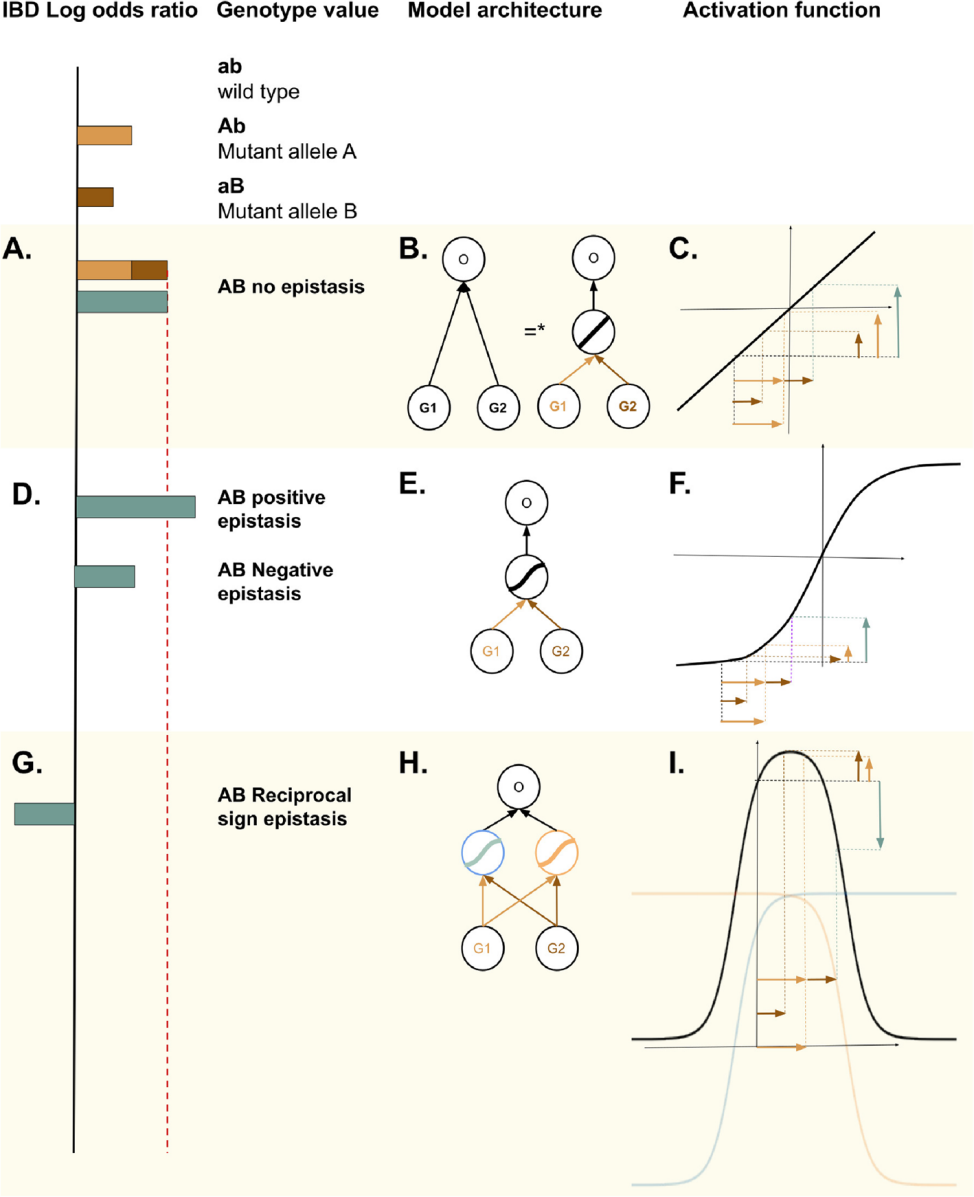
Different forms of biological epistasis (left) with minimal NN architectural requirements, illustrated by a small NN with two gene neuron inputs (middle) and the activation function of the hidden layer on top of the gene neurons (right). *A linear function of a linear function is a linear function

Nonlinear interactions between alleles, called epistasis, are ubiquitous at the molecular level [5, 11–17]. As shown in Fig. 3D, G, epistasis between two alleles at two loci (genes, in our case) means that the phenotypic effect of the allele at Locus 1 depends on the allele present at Locus 2 [15], thus deviating from a situation in which each locus independently influences the phenotype (additivity, see Fig. 3A). In positive and negative epistasis (Fig. 3D), the epistatic loci are affecting the magnitude of each other’s effect on the phenotype. Conversely, when two alleles alone have a negative phenotypic effect, but taken together they have a positive (or vice versa) effect (see lock-and-key model example in Additional file 6: Fig. S2), this is called reciprocal sign epistasis (Fig. 3G). Although our NNs produce individual-specific probability-like predictions, for illustrative purposes in Fig. 3, we associate log odds ratios to the hypothetical alleles shown, because we needed a metric that can be negative (i.e., indicating a protective effect of variants) to more clearly illustrate reciprocal sign epistasis.

In many phenotypes, especially in polygenic diseases such as IBD, the interactions between multiple genetic factors can contribute to the phenotype and the modulation of its severity [16, 17, 45, 46]. Therefore, it is unsurprising that, if the bias–variance trade-off allows a nonlinear model to robustly detect these patterns, this nonlinear model will outperform additive ones.

#### Relating the expressivity of NN architectures to epistasis types

From a mathematical perspective, a peculiarity of our NN_biosparse_ model (see Fig. 1B) is that the arrangement of the sparse interactions between the gene neurons *G* and the pathway neurons *P* is less expressive than a fully connected (dense) layer (see NN_dense_ in Fig. 1C), and therefore, it cannot model every pattern that is accessible to NN_dense_. To draw biological conclusions from this observation coupled with the observed prediction performances shown in Table 1, we link the expressivity of the three different NN architectures (i.e., the types of nonlinear patterns they can model) with the different forms of biological epistasis that they can detect (see Additional file 7: Table S4).

NN_logreg_ (Fig. 1A) is only capable of modeling additivity between genes, because no interaction between features (gene neurons *G* in this case) is allowed in their architecture (see Fig. 3A–C). The sparse architecture of NN_biosparse_, with the gene–pathway layer (see Fig. 1B) can model negative and positive epistasis between genes, because genes of the same pathway are allowed to interact via that pathway neuron, which has a nonlinear activation function (see Fig. 3E, F). Since our NN_biosparse_ design particularly allows for only one connection from each gene neuron to the next layer, two genes will never interact in more than one pathway neuron, making it impossible to model *nonlinearly separable patterns*, such as reciprocal sign epistasis. A fully connected layer such as in model in Fig. 1C, however, can model such epistasis, because it can model arbitrarily complex functions, including nonlinearly separable ones, as a *composition* of nonlinear activations (see Fig. 3I).

The last column of Fig. 3 illustrates an intuitive explanation of this behavior, by showing the output of three minimal examples of NNs representing our three architectures, having just two gene neurons as input. With a linear activation (see Fig. 3C), the joint effect of the two genes will always be additive, namely the simple sum of their separate effects, making it impossible to model epistatic interaction. However, if we add a nonlinear activation, such as $\tanh$, to the same model (Fig. 3F), the same shift on the *x*-axis can cause different shifts on the *y*-axis, depending where on the *x*-axis we are located. In Fig. 3F, we show an example of how such a model could learn a positive epistatic interaction between two genes, where the effect of the two genes together is larger than the sum of their separate effects. Nevertheless, $\tanh$ is a monotonically increasing function, making it impossible to model reciprocal sign epistasis, because the *joint* effect of both inputs cannot have opposite direction with respect to their separate effects. To allow this, we will need to break the monotonicity of the curve by allowing the NN architecture to learn how to compose the activation functions of more than one hidden neuron, thus needing more than one hidden neuron via which the genes can interact (see Fig. 3I). Intuitively, reciprocal sign epistasis is similar to the classical XOR classification problem in ML [47], that also needs at least two hidden neurons, representing an OR and NOT AND gate, to be modeled (see Additional file 6: Fig. S2).

#### Reciprocal sign epistasis is not detectable on the current dataset

From the performance in Table 1, we see that, given the currently available sample size, there is no advantage in investing more parameters (higher model complexity) towards training NNs capable of modeling reciprocal sign epistasis (NN_dense_) on this IBD cohort, since the significant increased model complexity produces lower AUC. Given the currently available samples in this IBD cohort, a model expressivity sufficient to address positive/negative epistasis seems therefore to be optimal. Similarly to what we previously discussed between additive and nonlinear models, the explanation for this fact may be that either (1) reciprocal sign epistasis does not have a major role in IBD pathogenesis or (2) the current sample size is insufficient to detect it.

To empirically verify that this inability is not just the result of the over-parametrization of NN_dense_ with respect to the dataset size, causing a suboptimal positioning in the bias–variance spectrum, we ran an additional experiment building two additional biologically sparsified architectures. The first architecture, shown in Additional file 8: Fig. S3A, has an identical number of neurons as NN_biosparse_ but allows for more than one connection for each gene, representing all the gene-pathway relations present in the KEGG database [40]. The second additional model, shown in Additional file 8: Fig. S3B, contains an extra sparse layer that mimics known gene–gene interactions from the Interactome database [48] before the gene–pathway layer. In both models each pair of genes can possibly reach the output neuron following more than one path (see Fig. 3H), thereby achieving the requirements necessary to model reciprocal sign epistasis as well. Notwithstanding the lower number of parameters compared to NN_dense_ (see Additional file 7: Table S4), none of these models outperform NN_biosparse_ (with mean (std) ROC AUC 0.756 (0.00829) and 0.729 (0.0110), respectively), suggesting that indeed either reciprocal sign epistasis is not present or not robustly detectable at this sample size. It will be interesting to further investigate these more complex architectures in future larger cohorts.

### Random sparsity outperforms biologically meaningful and learned sparsity

**Fig. 4:**
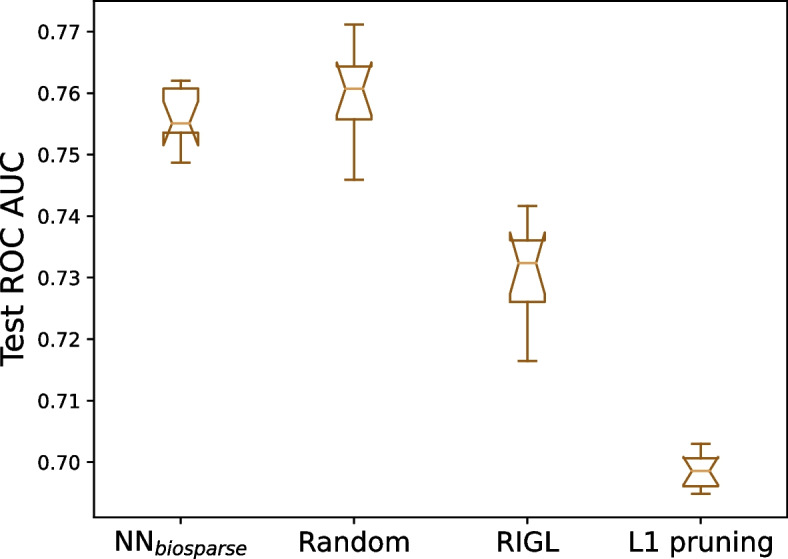
Comparison of four sparsification methods: **A** based on biological pathways (KEGG), **B** randomly, **C** learned using RigL algorithm, and **D** learned using heavy $\text {L}_1$ regularization. For fair comparison, all models shown have the same number of connections and hidden units

Table 1 shows that the sparse gene–pathway layer in our NN_biosparse_ is instrumental towards its performance, because it allows an optimal level of expressivity while avoiding over-parametrization. But does the biologically meaningful arrangement of these connections between neurons mimicking genes and pathways also play a role towards prediction accuracy? In Fig. 4, we compare the performance of the knowledge-based sparsity pattern extracted from KEGG with three other connection arrangements using the same number of connections, thus without increasing the number of parameters.

The first surprising result is that the knowledge-based sparsification does not outperform the random one (see Fig. 4). Using biological networks to sparsify gene–pathway interactions in an NN layer seemed an obvious choice, since it assumed that genes participating in the same pathway could be more prone towards modulating each other in an epistatic fashion. However, databases such as KEGG are still far from complete: only one third of the genes in our dataset could be connected to a KEGG pathway (as described in the “Material and methods” section, we joined them to a “dummy neuron” to avoid discarding them).

In Fig. 4, we also benchmarked two ways to *learn* optimal sparsity arrangements from the data, using the RigL [49] method and imposing $L_1$ regularization on the gene–pathway connections. In both cases, the learned sparsity yielded lower performance. We hypothesize that by learning which connections to make and thus optimizing sparsity during training, we offer the model another way to overfit the noisy training data, in contrast to the strong regularizing effect of random sparsity. For more details on the RigL method and its benchmark settings, see Additional file 9: Fig. S4 and Additional file 10: Suppl. Method.

**Fig. 5:**
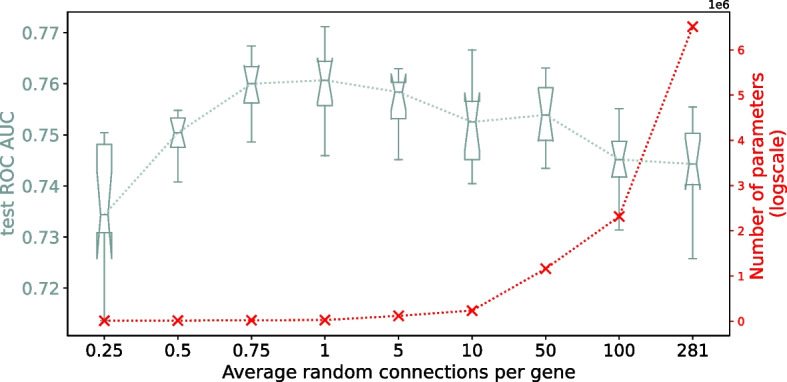
Effect of degree of sparsity on performance, going from only 25% of the genes randomly connected to a fully connected layer. Red crosses indicate the number of parameters for each model

Since the prediction performance does not seem to benefit from learned or biologically meaningful arrangements of the hidden layer sparsity, we investigate whether the *level* of sparsity is playing a more important factor role. In Fig. 5, we compare the performance of several random sparsity degrees, ranging from only connecting 25% of the genes to a pathway hidden neuron, to a fully connected layer with 281 connections for each gene. The figure shows an optimum at 75% of the genes connected, suggesting that the model could be regularized even further by dropping some genes completely (although the difference in performance for 75% and 100% of the genes connected is not statistically significant).

This shows that irrespectively of the *arrangement* of the connections, the main driver of the model performance is the degree of sparsity, and the effect it has on its complexity (i.e., its position in the bias–variance spectrum).

### Further exploiting random connections with ensemble learning leads to the best predictions

Given the results obtained by random sparsification in our models, we decided to exploit this behavior to build an ensemble learner of randomly sparsified NNs to solve the IBD prediction task. Ensemble methods follow the intuition that combining a set of (possibly weak) base learners with low correlation between them will lead to better prediction, more robust to noise [50]. The crucial step in this process is minimizing the correlation between the base learners by injecting a random element in each of them. Conventionally, the randomness is introduced through features and input sample subsampling. Here, we randomized instead the connection arrangements in our sparse NN layer. We thus train 100 randomly sparsified NNs on the full training set and average their prediction. In Table 2, we show that the ensemble model has an almost 2% increase in ROC AUC with respect to NN_biosparse_ (corrected two-sided *t*-test [42] $p=0.04146$).

**Table 2:** Ensemble model

Model	ROC AUC^a^	Parameters
NN_biosparse_	0.758 (0.00689)	25,503
Randomly sparsified (100% genes connected)	0.759 (0.00778)	25,503
Randomly sparsified (75% genes connected)	0.760 (0.00571)	19,708
Ensemble (100% genes connected)	0.774 (0.00276)	25,503
Ensemble (75% genes connected)	0.776 (0.00265)	19,709

^a^Performance given as mean (standard deviation) of test set ROC AUC from 10 different full threefold cross-validation runs

For completeness, we performed additional experiments aiming at predicting Crohn’s disease and ulcerative colitis, the two main IBD subtypes, separately, notwithstanding the consequently smaller sample sizes available for training (see Additional file 11: Fig. S5, Additional file 12: Table S5, Additional file 13: Note S2).

## Conclusions

In this article, we zoomed in on the role that the widespread underdetermination of genetic datasets ($p\ll n$) plays on the performance of different modeling methods, depending on their position in the bias–variance spectrum. We thereby showed that underdetermination is plausibly one of the major drivers for the apparent optimality of additive modeling in clinical genetics today. We showed that when we reach more favorable *n*/*p* ratios by constraining the NN complexity and providing enough training data, NNs outperform conventional additive models in WES-based IBD case–control discrimination. To do so, we proposed a biologically meaningful sparsified NN architecture, but further experiments showed that the degree of sparsity is more decisive for predictive performance than the biological meaningfulness of the connections, again emphasizing the importance of underdetermination. Furthermore, by linking the expressivity of our NN architectures with different forms of biological epistasis, we provided empirical evidence that positive/negative epistatic effects are present in the genetic architecture of IBD. Our result suggests that larger cohorts will allow further improvement through more complex modeling architectures in the near future, thereby enabling a new, nonadditive *lens* on genome interpretation and contributing to a more complete molecular picture of all kinds of phenotypes.

## Materials and methods

### Dataset

We analyzed the data from the inflammatory bowel disease (IBD) exome sequencing study (dbGaP phs001076.v1.p1) [51], a case–control study containing whole exome sequences (WES) for 3318 IBD cases and 480 controls. The 3318 cases consist of the two main subtypes of IBD: 2036 Crohn’s disease (CD) patients and 1215 ulcerative colitis (UC) patients. For 67 cases, the IBD subtype is unknown (indeterminate colitis). In the control group, 39.4% of the participants are male compared to 46.7% of the cases. The data is provided as a VCF file listing the observed variants.

### Encoding the exome variants into feature vectors

Encoding WES data into ML-ready feature vectors is not trivial, since each individual carries an arbitrary number of variants (on average 47,403 in our dataset), and the entire dataset covers a large pool of variants (1,733,480 unique variants in our dataset). To overcome this issue, we followed the approach we adopted in our previous works [36, 37], which we briefly summarize here.

We first annotated all variants in the VCF with Annovar [52], assigning each of them to a gene and to one of the following 16 functional classes: UTR3, UTR5, splicing, upstream, downstream, intronic, intronic ncRNA, exonic ncRNA, splicing ncRNA, exonic non-frameshift insertion, exonic frameshift insertion, exonic non-frameshift deletion, exonic frameshift deletion, exonic stoploss, exonic stopgain, exonic startloss and exonic nonsynonymous. We then summarized the annotated variants by aggregating them at the gene level [36, 37], making the gene the base semantic entity in our feature encoding. To do so, we count for each gene how many variants of a specific functional class map to it. In this way, each gene’s *mutational load* is quantified by a 16-dimensional histogram. Each WES sample is thus described by a matrix of size $16 \times 23,177$ with 23,177 being the total number of genes in the dataset. We refer to this representation as *gene-centric encoding* [36, 37]. It is possible to further enrich this representation using gene-level and variant-level scores as additional features [37]. To avoid numerical issues, we standardized the counts of each type of variant across all genes.

### A biologically meaningful sparse NN architecture for GI

#### A shared gene module decouples the number of parameters from the number of genes

After defining our gene-centric feature encoding for WES data, we describe the NN architectures we devised to process this data and address the $p \gg n$ issue. Similarly to our previous work [36, 37], we process each gene histogram in our gene-centric encoding with a shared gene module (see Fig. 1). By sharing the same NN module across all the genes, we minimize the number of parameters needed to transform the (16, 23177) input matrix representing each sample into a compact latent vector of length 23,177, where each gene is described by just 1 value (the output of the shared gene module, mentioned as neuron $G_i$ for the $i^{th}$ gene, with $1\le i \le 23,177$ and the total set of gene neurons being $G = \{G_1, \dotsc , G_{23,177}\}$). In all the models presented here, we used the same architecture for this shared gene module, with 1 hidden layer of 50 neurons (see Fig. 1)) (chosen as the smallest architecture needed to perfectly fit the training data after a coarse grained tuning phase), which brought the total number of trainable parameters to a modest 850 weights plus 51 biases.

#### Exploiting the small-worldness of biological networks to sparsify the NN architecture

Figure 1 illustrates the three different NN achitecture prototypes built on top of the shared gene module described above. The simplest approach connects the $|G| = 23,177$ neurons to the output prediction, implementing a logistic regression (LogReg) of the gene neuron activations (see Fig. 1A). We refer to this model as NN_logreg_. Adding a dense hidden layer (see Fig. 1C) between the gene layer and the output allows us to surpass the limited expressivity of the simple LogReg and to capture nonlinear interactions between the genes, but at the same time it causes a combinatorial explosion of the number of parameters. We refer to this model as NN_dense_.

To obtain the best of both worlds, meaning nonlinear inference and maximal parameter reduction, in Fig. 1B, we sparsify the connections between the |*G*| neurons and the next layer. To do so in a *biologically meaningful* manner, we randomly pick for each gene one of the KEGG pathways it contributes to [40]. Two thirds of the genes in the dataset (15,219 out of 23,177 genes) do not belong to any known KEGG pathway and they were connected to one *dummy* pathway neuron, to avoid discarding them. The idea behind this architecture is that we assign a biological meaning to each NN module, since each neuron in Fig. 1B represents a biological unit, such as a Gene $G_i$ or a Pathway $P_j$. This way we preserve the interpretability of the model, while requiring significantly fewer parameters than an NN with a dense hidden layer (Fig. 1B). We refer to this model as the biologically sparsified NN (NN_biosparse_).

Biologically sparsified layers, like any other NN module, can be stacked on top of each other. For example, in Additional file 8: Fig. S3, we built an NN with two NN_biosparse_ layers, mimicking gene–gene [48, 53] and gene–pathway interactions, thus allowing even more complex interactions between neurons, and thus a more expressive NN model, at the cost of an increased number of parameters, yet still lower than NN_dense_.

### Implementation details

All NNs in this paper have been implemented with PyTorch [54] and are trained with the Adam optimizer, a learning rate of 0.001, a batch size of 128, and a weighted focal binary cross-entropy loss function ($\alpha = 480/(480+3,318) = 0.12638$, $\gamma = 2$) to address the class imbalance. Weight decay and dropout layers were used as regularization techniques. Epochs ranged between 20 and 75 depending on the model architecture. The hyperparameter search grid for the different models is supplied in Additional file 14: Table S6. The source code is freely available at https://bitbucket.org/noraver/ibd_gi/src/master/ [55]. All models in this paper were evaluated using 10 repetitions of threefold cross-validation with stratified splits. We report the mean and standard deviation of the area under the ROC curve (AUC) of the test set. The same splits were used to compare models in Table 1 and Figs. 2, 4, and 5. The choice to use three folds was motivated by computational time. To verify that the same patterns occur in other cross-validation approaches, a tenfold cross-validation was performed for the best linear model and neural network, with results shown in Additional file 15: Table S7. The performances on the individual cross-validation runs of Fig. 2 are shown in more detail in Additional file 16: Fig. S6.

### Baseline additive and nonlinear models

We used logistic regression with $L_1$ and $L_2$ penalty as additive baseline models. We implemented it using log loss with the SGDClassifier from the scikit-learn library [56]. As they were applied to the individual variants, these models have 1,733,481 parameters and needed regularization ($\alpha = 1$ for $L_2$ penalty, $\alpha = 0.01$ for $L_1$ penalty). As a nonlinear baseline method, we used the Random Forest (RF) model from scikit-learn. The RF on the individual variants uses the 1,733,480 dimensional feature vector described above, it has 1000 estimators of a maximum depth of 1000. The RF on the summed gene vectors uses as input the sum of the above-described 16-dimensional *mutational load* histograms for each gene and 10,000 estimators of a maximum depth of 3. The RF on the NN learned gene activations uses the gene activation values extracted from the first hidden layer of the fully trained NN_logreg_, NN_biosparse_ or NN_dense_ as input, and 10,000 estimators of a maximum depth of 3.

### Supplementary information

**Additional file 1: Figure S1.** Inclusion of only known IBD genes.

**Additional file 2: Table S1.** Inclusion of gene- and variant-level scores in input representation.

**Additional file 3: Table S2.** Minor allele frequency (MAF) based preselection.

**Additional file 4: Note S1.** Analysis of GWAS variants.

**Additional file 5: Table S3.** Random Forest Classifier performances on different data representations.

**Additional file 6: Figure S2.** Exclusive OR (XOR) as a well known nonlinearly separable problem in machine learning.

**Additional file 7: Table S4.** Model overview with number of parameters and interaction patterns that can be captured.

**Additional file 8: Figure S3.** Biologically sparsified model with gene-gene interaction and gene-pathway layer.

**Additional file 9: Figure S4.** Benchmarking of sparsification using Rigl and random connections.

**Additional file 10.** Supplementary Method: Rigl.

**Additional file 11: Figure S5.** Subtype analysis: additive versus neural network on different subsamples.

**Additional file 12: Table S5.** Subtype analysis on Crohn’s Disease and Ulcerative Colitis.

**Additional file 13: Note S2.** Subtype analysis on Crohn’s Disease and Ulcerative Colitis.

**Additional file 14: Table S6.** Model hyperparameters.

**Additional file 15: Table S7.** Tenfold cross-validation performance of best linear model and neural net.

**Additional file 16: Figure S6.** Details of individual cross-validation runs for Fig. 2 on the performance of different random subsets containing 10%, 20%, 40%, 60%, 80%, and 100% of the dataset.

**Additional file 17.** Review history.

## Data Availability

All the code described in this paper is available at https://bitbucket.org/noraver/ibd_gi/src/master/ [55]. This program is free software; you can redistribute it and/or modify it under the terms of the GNU General Public License as published by the Free Software Foundation; either version 2 of the License, or any later version. No custom scripts and software were used other than those mentioned in the “Material and methods” section. The code is deposited in Zenodo as well with DOI 10.5281/zenodo.8324997 [57]. The data that support the findings of this study are available from dbGaP (Inflammatory Bowel Disease Exome Sequencing Study, dbGaP Study Accession: phs001076.v1.p1) but restrictions apply to the availability of these data. Access to the data can be requested through dbGaP [51].
