# Supplementary material for: Large sample size and nonlinear sparse models outline epistatic effects in inflammatory bowel disease
Source: Genome Biol. 2023 Oct 5;24:224. doi: 10.1186/s13059-023-03064-y (PMC10552306; doi:10.1186/s13059-023-03064-y)
Supplement: Supplementary file 1 — Additional file 1: Figure S1. Inclusion of only known IBD genes. [file 13059_2023_3064_MOESM1_ESM.pdf]

Additional file 1: Fig. S1: Inclusion of only known IBD genes

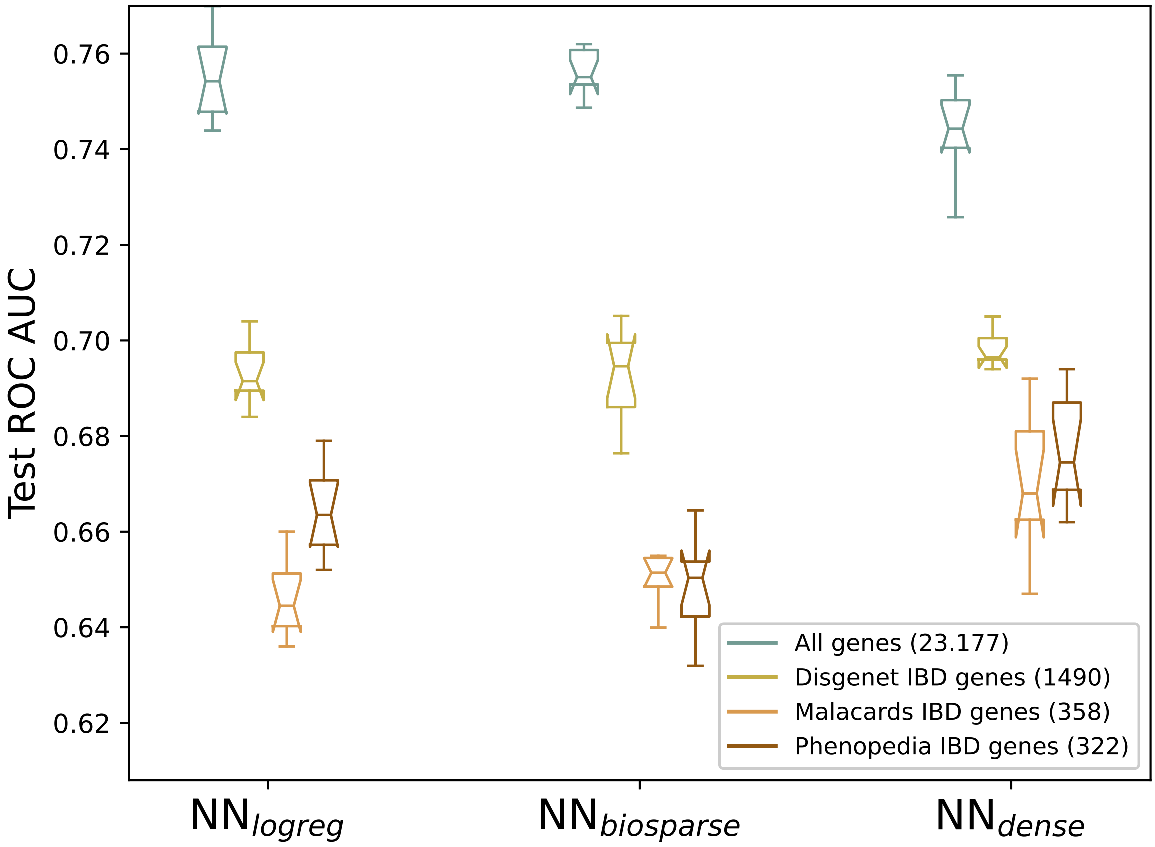

Figure 1: Effect on performance of keeping only known IBD genes for the main model types (NN_{logreg}, $NN_{biosparse}$, NN_{dense}). Three different databases were used to select the IBD genes, resulting in 1490 IBD genes from Disgenet [1], 358 IBD genes from Malacards [2] and 323 IBD genes from Phenopedia [3].

References

[1] Piñero, J., Ramírez-Anguita, J.M., Saüch-Pitarch, J., Ronzano, F., Centeno, E., Sanz, F., Furlong, L.I.: The DisGeNET knowledge platform for disease genomics: 2019 update. Nucleic Acids Research **48**(D1), 845–855 (2019)

[2] Rappaport, N., Twik, M., Plaschkes, I., Nudel, R., Iny Stein, T., Levitt, J., Gershoni, M., Morrey, C.P., Safran, M., Lancet, D.: MalaCards: an amalgamated human disease compendium with diverse clinical and genetic annotation and structured search. Nucleic Acids Research **45**(D1), 877–887 (2016)

[3] Yu, W., Clyne, M., Khoury, M.J., Gwinn, M.: Phenopedia and Genopedia: disease-centered and gene-centered views of the evolving knowledge of human genetic associations. Bioinformatics **26**(1), 145–146 (2009)
