# Supplementary material for: Large sample size and nonlinear sparse models outline epistatic effects in inflammatory bowel disease
Source: Genome Biol. 2023 Oct 5;24:224. doi: 10.1186/s13059-023-03064-y (PMC10552306; doi:10.1186/s13059-023-03064-y)
Supplement: Supplementary file 2 — Additional file 2: Table S1. Inclusion of gene- and variant-level scores in input representation. [file 13059_2023_3064_MOESM2_ESM.pdf]

Additional file 2: Table S1: Inclusion of gene- and variant-level scores in input representation

Input representation	ROC AUC
Functional gene element histograms only	0.758 (0.00689)
+ gene-level scores	0.740 (0.0218)
+ variant-level scores	0.730 (0.0141)
+ gene-level and variant-level scores	0.731 (0.00788)

*Performance in terms of test set ROC AUC, given for the $NN_{biosparse}$ model as mean and standard deviation from 10 different full threefold cross-validation runs. Gene-level scores included are Gene Damage Index (GDI) [1], Residual Variation Intolerance Score (RVIS) [2], Haploinsufficiency probability [3] and Recessive probability [4]. Variant-level scores included are Protein Variation Effect Analyzer (PROVEAN) [5], Mendelian Clinically Applicable Pathogenicity (M-CAP) [6], metaSVM [7] and DEOGEN2 [8].

References

[1] Itan, Y., Shang, L., Boisson, B., Patin, E., Bolze, A., Moncada-Velez, M., Scott, E., Ciancanelli, M., Lafaille, F., Markle, J., Martinez Barricarte, R., de Jong, S., Kong, X.-F., Nitschke, P., Belkadi, A., Bustamante, J., Puel, A., Boisson-Dupuis, S., Stenson, P., Casanova, J.-L.: The human gene damage index as a gene-level approach to prioritizing exome variants. Proceedings of the National Academy of Sciences of the United States of America **112** (2015)

[2] Petrovski, S., Wang, Q., Heinzen, E., Allen, A., Goldstein, D.: Genic intolerance to functional variation and the interpretation of personal genomes. PLoS Genet. **9** (2013)

[3] Huang, N., Lee, I., Marcotte, E., Hurles, M.: Characterising and predicting haploinsufficiency in the human genome. PLoS genetics **6**, 1001154 (2010)

[4] Macarthur, D., Balasubramanian, s., Frankish, A., Huang, N., Morris, J., Walter, K., Jostins, L., Pickrell, J., Montgomery, S., Albers, C., Zhang, Z., Conrad, D., Lunter, G., Zheng, H., Ayub, Q., DePristo, M., Banks, E., Hu, M., Tyler-Smith, C.: A systematic survey of loss-of-function variants in human protein-coding genes. Science (New York, N.Y.) **335**, 823–8 (2012)

[5] Choi, Y., Chan, A.: Provean web server: A tool to predict the functional effect of amino acid substitutions and indels. Bioinformatics (Oxford, England) **31** (2015)

[6] Jagadeesh, K., Wenger, A., Berger, M., Guturu, H., Stenson, P., Cooper, D., Bernstein, J., Bejerano, G.: M-cap eliminates a majority of variants of uncertain significance in clinical exomes at high sensitivity. Nature genetics **48** (2016)

[7] Liu, X., Wu, C., Li, C., Boerwinkle, E.: dbnsfp v3.0: A one-stop database of functional predictions and annotations for human non-synonymous and splice site snvs. Human mutation **37** (2015)

[8] Raimondi, D., Tanyalçin, I., Ferte, J., Gazzo, A., Orlando, G., Lenaerts, T., Rooman, M., Vranken, W.: Deogen2: prediction and interactive visualization of single amino acid variant deleteriousness in human proteins. Nucleic acids research **45** (2017)
