# Supplementary material for: Large sample size and nonlinear sparse models outline epistatic effects in inflammatory bowel disease
Source: Genome Biol. 2023 Oct 5;24:224. doi: 10.1186/s13059-023-03064-y (PMC10552306; doi:10.1186/s13059-023-03064-y)
Supplement: Supplementary file 3 — Additional file 3: Table S2. Minor allele frequency (MAF) based preselection. [file 13059_2023_3064_MOESM3_ESM.pdf]

Additional file 3: Table S2: Minor allele frequency (MAF) based preselection

MAF selection	% variants included	ROC AUC
$< 0.1\%$	79.04%	0.573(0.0523)
$< 1\%$	91.45%	0.606 (0.0315)
$< 5\%$	94.62%	0.652 (0.0206)
$< 10\%$	95.7%	0.667 (0.0369)
$< 50\%$	98.6%	0.714 (0.0128)
$> 0.1\%$	20.09%	0.740 (0.0115)
$> 1\%$	8.48%	0.740 (0.00919)
$> 5\%$	5.34%	0.740 (0.0109)
$> 10\%$	4.25%	0.744 (0.00572)
$> 50\%$	1.36%	0.720 (0.0108)
0.1-5%	15.58%	0.740 (0.0117)
1-5%	3.17%	0.607 (0.0120)

*Performance in terms of test set ROC AUC, given for the $NN_{biosparse}$ model as mean and standard deviation from 10 different full threefold cross-validation runs.
