# Supplementary material for: Large sample size and nonlinear sparse models outline epistatic effects in inflammatory bowel disease
Source: Genome Biol. 2023 Oct 5;24:224. doi: 10.1186/s13059-023-03064-y (PMC10552306; doi:10.1186/s13059-023-03064-y)
Supplement: Supplementary file 4 — Additional file 4: Note S1. Analysis of GWAS variants. [file 13059_2023_3064_MOESM4_ESM.pdf]

Additional file 4: Note S1: Analysis of GWAS variants

In an attempt to add Polygenic Risk Scores (PRS) to our benchmark, we calculated for each Inflammatory Bowel Disease (IBD) PRS identified in the PGS catalog [1, 2, 3] the percentage of variants present in our WES IBD dataset. From the PRS with the most overlapping variants [3], only 42% of the variants were present in our dataset, resulting in a total of 82 variants. We computed a PRS score for each sample using a linear model of these 82 variants with the coefficients derived from the odds ratios from the corresponding genome-wide association study. This resulted in a classifier with a ROC AUC of 0.563. Other linear and nonlinear models built with these 82 selected variants as input did not significantly improve on the performance, as shown in the table below.

Model	ROC AUC*	Tuned hyperparameters
PRS with GWAS odds ratios	0.563 (0.0)	
L2 penalized linear model (Ridge)	0.546 (0.0104)	$alpha = 1000$
L1 penalized linear model (Lasso)	0.546 (0.0108)	$alpha = 1$
Random Forest Classifier	0.553 (0.0102)	max_depth = 3, n_estimators = 10000
Neural network Classifier	0.536 (0.0180)	1 hidden layer with 10 neurons and ReLU activation
		$alpha = 0.000001$

* Performance given as mean (standard deviation) of test set ROC AUC from ten different full threefold cross-validation runs with the same fold splits for all models.

References

[1] Lambert, S., Gil, L., Jupp, S., Ritchie, S., Xu, Y., Buniello, A., Abraham, G., Chapman, M., Parkinson, H., Danesh, J., MacArthur, J., Inouye, M.: The Polygenic Score Catalog: an Open Database for Reproducibility and Systematic Evaluation

[2] Khera, A., Chaffin, M., Aragam, K., Haas, M., Roselli, C., Choi, S., Natarajan, P., Lander, E., Lubitz, S., Ellinor, P., Kathiresan, S.: Genome-wide polygenic scores for common diseases identify individuals with risk equivalent to monogenic mutations. Nature Genetics **50** (2018). doi:10.1038/s41588-018-0183-z

[3] Tanigawa, Y., Qian, J., Venkataraman, G., Justesen, J., Li, R., Tibshirani, R., Hastie, T., Rivas, M.: Significant sparse polygenic risk scores across 813 traits in uk biobank. PLOS Genetics **18**, 1010105 (2022)
