# Supplementary material for: Large sample size and nonlinear sparse models outline epistatic effects in inflammatory bowel disease
Source: Genome Biol. 2023 Oct 5;24:224. doi: 10.1186/s13059-023-03064-y (PMC10552306; doi:10.1186/s13059-023-03064-y)
Supplement: Supplementary file 5 — Additional file 5: Table S3. Random Forest Classifier performances on different data representations. [file 13059_2023_3064_MOESM5_ESM.pdf]

Additional file 5: Table S3: Random Forest Classifier performances on different data representations

Model	ROC AUC*
Random forest on individual variants	0.688 (0.00578)
Random forest on summed gene vectors	0.672 (0.00279)
Random forest on NN_{logreg} learned gene activations	0.588 (0.00471)
Random forest on $NN_{biosparse}$ learned gene activations	0.601 (0.0108)
Random forest on NN_{dense} learned gene activations	0.581 (0.00607)

* Performance given as mean (standard deviation) of test set ROC AUC from 10 different full threefold cross-validation runs with the same fold splits for all models.
