# Supplementary material for: Large sample size and nonlinear sparse models outline epistatic effects in inflammatory bowel disease
Source: Genome Biol. 2023 Oct 5;24:224. doi: 10.1186/s13059-023-03064-y (PMC10552306; doi:10.1186/s13059-023-03064-y)
Supplement: Supplementary file 6 — Additional file 6: Figure S2. Exclusive OR (XOR) as a well known nonlinearly separable problem in machine learning. [file 13059_2023_3064_MOESM6_ESM.pdf]

Additional file 6: Fig. S2: Exclusive OR (XOR) as a well known nonlinearly separable problem in machine learning

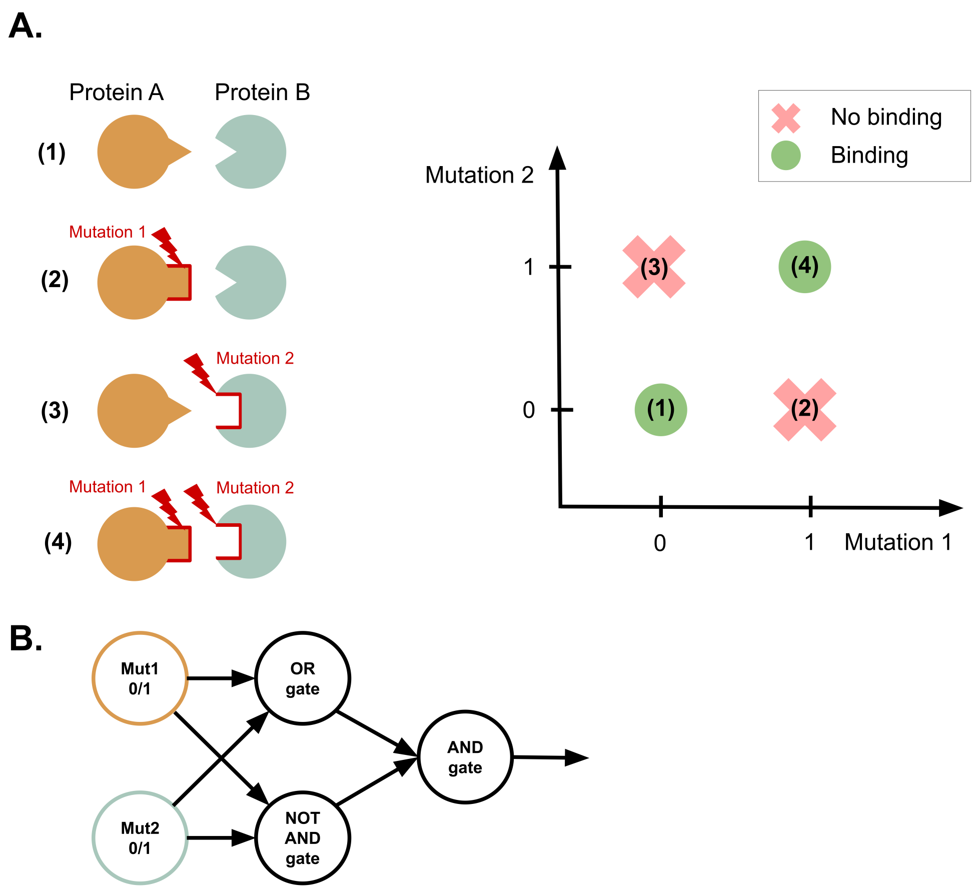

Figure 1: (A) Illustrative example of the nonlinear XOR interaction, a well known nonlinearly separable machine learning problem, in a biological pathway named *lock-and-key model*, which occurs between two mutations in the binding pocket of two proteins. (B) The minimal needed neural network architecture to model XOR classification, needing at least two hidden neurons, representing an OR and NOT AND operation.
