# Supplementary material for: Large sample size and nonlinear sparse models outline epistatic effects in inflammatory bowel disease
Source: Genome Biol. 2023 Oct 5;24:224. doi: 10.1186/s13059-023-03064-y (PMC10552306; doi:10.1186/s13059-023-03064-y)
Supplement: Supplementary file 7 — Additional file 7: Table S4. Model overview with number of parameters and interaction patterns that can be captured. [file 13059_2023_3064_MOESM7_ESM.pdf]

Additional file 7: Table S4: Model overview with number of parameters and interaction patterns that can be captured

Model	Number of parameters	Additive interaction	Negative/Positive epistasis	Reciprocal sign epistasis
Ridge regression	1.734.301	Yes	No	No
NN_{linear}	25.503	Yes	No	No
$NN_{biosparse}$	25.503	Yes	Yes	No
Biologically sparsified NN with all KEGG gene-pathway connections	52.868	Yes	Yes	Yes
Biologically sparsified NN with additional gene-gene interaction layer	219.861	Yes	Yes	Yes
