# Supplementary material for: Large sample size and nonlinear sparse models outline epistatic effects in inflammatory bowel disease
Source: Genome Biol. 2023 Oct 5;24:224. doi: 10.1186/s13059-023-03064-y (PMC10552306; doi:10.1186/s13059-023-03064-y)
Supplement: Supplementary file 8 — Additional file 8: Figure S3. Biologically sparsified model with gene-gene interaction and gene-pathway layer. [file 13059_2023_3064_MOESM8_ESM.pdf]

Additional file 8: Fig. S3: Additional biologically sparsified models with more connections and expressivity

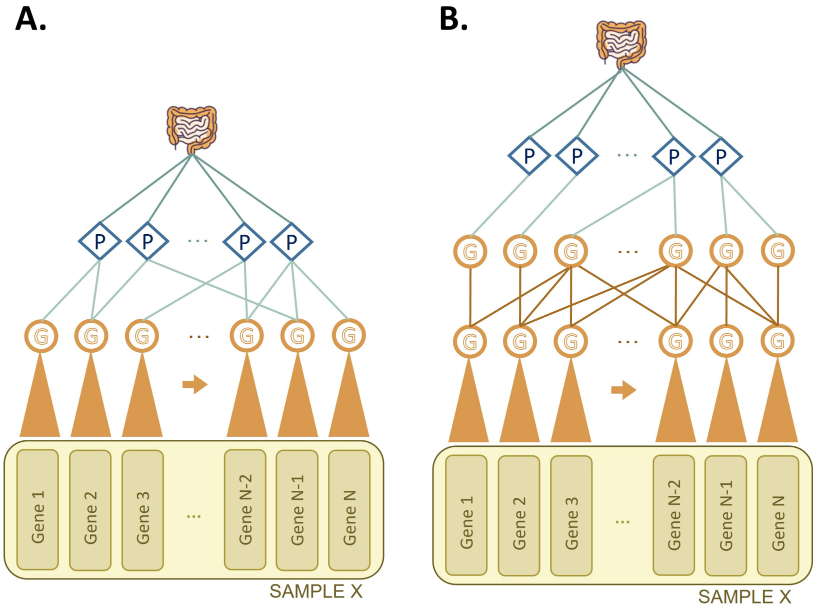

Figure 1: Additional biologically sparsified models with (A) containing identical neurons as $NN_{biosparse}$ but all gene-pathway connections in the KEGG database, and (B) containing an additional sparse gene-gene interaction layer before the gene-pathway layer.
