# Supplementary material for: Large sample size and nonlinear sparse models outline epistatic effects in inflammatory bowel disease
Source: Genome Biol. 2023 Oct 5;24:224. doi: 10.1186/s13059-023-03064-y (PMC10552306; doi:10.1186/s13059-023-03064-y)
Supplement: Supplementary file 9 — Additional file 9: Figure S4. Benchmarking of sparsification using Rigl and random connections. [file 13059_2023_3064_MOESM9_ESM.pdf]

Additional file 9: Fig. S4: Benchmarking of sparsification using Rigl and random connections

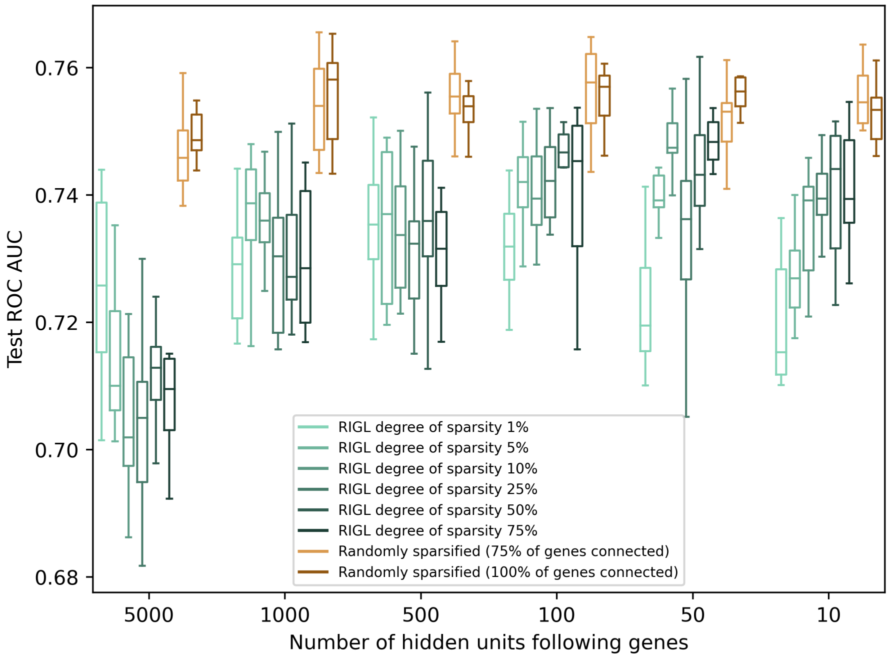

Figure 1: Benchmarking of different degrees of sparsity and hidden units for the Rigl method and the randomly sparsified model.
