# Supplementary material for: Large sample size and nonlinear sparse models outline epistatic effects in inflammatory bowel disease
Source: Genome Biol. 2023 Oct 5;24:224. doi: 10.1186/s13059-023-03064-y (PMC10552306; doi:10.1186/s13059-023-03064-y)
Supplement: Supplementary file 10 — Additional file 10. Supplementary Method: Rigl. [file 13059_2023_3064_MOESM10_ESM.pdf]

Additional file 10: Supplementary Methods: RigL

RigL is a recently proposed method to learn sparse networks with a fixed computational cost, by performing topology updates of the sparse network during training using parameter magnitudes and infrequent gradient calculations [1]. By avoiding the typical approach of pruning, where one starts with the fully connected layer and gradually cuts down connections, computational and memory cost can be limited for large NN layers.

References

[1] Evci, U., Gale, T., Menick, J., Rivadeneira, P.S.C., Elsen, E.: Rigging the lottery: Making all tickets winners. In: International Conference of Machine Learning (2020)
