# Supplementary material for: Large sample size and nonlinear sparse models outline epistatic effects in inflammatory bowel disease
Source: Genome Biol. 2023 Oct 5;24:224. doi: 10.1186/s13059-023-03064-y (PMC10552306; doi:10.1186/s13059-023-03064-y)
Supplement: Supplementary file 11 — Additional file 11: Figure S5. Subtype analysis: additive versus neural network on different subsamples. [file 13059_2023_3064_MOESM11_ESM.pdf]

Additional file 11: Fig. S5: Subtype analysis: additive versus neural network on different subsamples

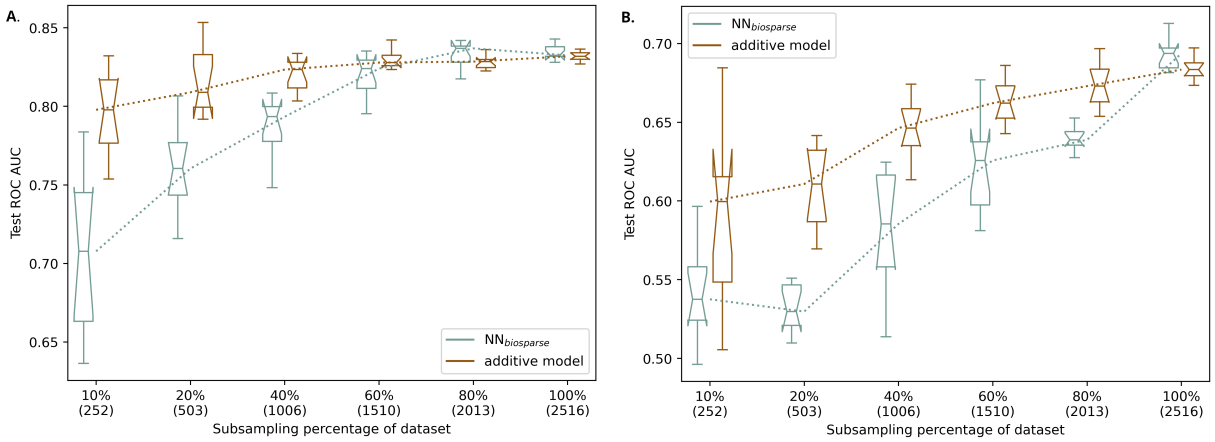

Figure 1: Performance using different random subsets containing 10%, 20%, 40%, 60%, 80% and 100% of the dataset in (A) for Crohn's disease versus control and in (B) for Ulcerative colitis versus control. Models shown are the best additive model (L_2 penalty) and $\mathrm{NN}_{\mathrm{biosparse}}$.
