# Supplementary material for: Large sample size and nonlinear sparse models outline epistatic effects in inflammatory bowel disease
Source: Genome Biol. 2023 Oct 5;24:224. doi: 10.1186/s13059-023-03064-y (PMC10552306; doi:10.1186/s13059-023-03064-y)
Supplement: Supplementary file 12 — Additional file 12: Table S5. Subtype analysis on Crohn’s Disease and Ulcerative Colitis. [file 13059_2023_3064_MOESM12_ESM.pdf]

Additional file 12: Table S5: Subtype analysis on Crohn's Disease and Ulcerative Colitis

Model	Crohn's disease versus control	Ulcerative colitis versus control
Ridge regression	0.833 (0.00388)	0.682 (0.00912)
$NN_{biosparse}$	0.833 (0.00747)	0.690 (0.0173)
Randomly sparsified (100% of genes connected)	0.830 (0.00571)	0.690 (0.0194)
Randomly sparsified (75% of genes connected)	0.827 (0.00316)	0.689 (0.0121)
Ensemble (100% of genes connected)	0.847 (0.00253)	0.726 (0.00726)
Ensemble (75% of genes connected)	0.845 (0.00229)	0.727 (0.00639)

*Performance given as mean (standard deviation) of 10 full runs of threefold cross-validation
