# Supplementary material for: Large sample size and nonlinear sparse models outline epistatic effects in inflammatory bowel disease
Source: Genome Biol. 2023 Oct 5;24:224. doi: 10.1186/s13059-023-03064-y (PMC10552306; doi:10.1186/s13059-023-03064-y)
Supplement: Supplementary file 13 — Additional file 13: Note S2. Subtype analysis on Crohn’s Disease and Ulcerative Colitis. [file 13059_2023_3064_MOESM13_ESM.pdf]

Additional file 13: Note S2: Subtype analysis on Crohn's Disease and Ulcerative Colitis

IBD can be divided into two main subgroups, Crohn's Disease (CD) and Ulcerative Colitis (UC). They share a significant portion of their genetic mechanisms, demonstrated by the overlap of the majority of their associated GWAS loci (160 out of 240) [1, 2]. For up to 10% of the IBD cases, a clinical and pathological distinction between either CD or UC cannot be made, and they are classified as "undetermined colitis" [3]. These cases constitute 2% of our dataset. This unclear distinction, together with the genetic overlap and also genetic heterogeneity within the subtypes themselves [4, 5], suggests a more continuous disease spectrum for IBD [1]. For completeness, we performed additional experiments aiming at predicting the two subtypes separately, notwithstanding the consequently smaller sample sizes available for training.

Suppl. Table 5 shows that when predicting CD and UC separately, $\mathrm{NN}_{\mathrm{biosparse}}$ could not significantly outperform the best additive model. In Suppl. Fig. 6, we tried to replicate the experiment shown in Fig. 2 to test the effect of sample size on the relative performance of both models. When we look at the trajectories in Suppl. Fig. 6, we see the same trend as before, suggesting that a cross-over could happen at larger sample size. Unfortunately, when we subsample the original dataset to create CD and UC datasets, the sample sizes n_{UC} and n_{CD} fall under the threshold t that we identified previously as needed to witness the advantage of the nonlinear model. Nevertheless, even at this sample size, ensemble learning with the randomly sparsified NN models significantly outperforms the additive baseline for both phenotypes (two-sided t-test $p = 1.120\mathrm{e}{-08}$ for CD and $p = 2.379\mathrm{e} - 10$ for UC, see Suppl. Table 5 for more details). This subgroup analysis confirms the role of sample size in the additive versus nonlinear modeling conundrum.

References

[1] Ellinghaus, D., Bethune, J., Petersen, B.-S., Franke, A.: The genetics of crohn's disease and ulcerative colitis – status quo and beyond. Scandinavian Journal of Gastroenterology **50**(1), 13–23 (2015). PMID: 25523552

[2] Lange, K., Moutsianas, L., Lee, J., Lamb, C., Luo, Y., Kennedy, N., Jostins, L., Rice, D., Gutierrez-Achury, J., Ji, S.-G., Heap, G., Nimmo, E., Edwards, C., Henderson, P., Mowat, C., Sanderson, J., Satsangi, J., Simmons, A., Wilson, D., Barrett, J.: Genome-wide association study implicates immune activation of multiple integrin genes in inflammatory bowel disease (2016)

[3] James, S., Wise, P., Zuluaga-Toro, T., Schwartz, D., Washington, M., Shi, C.: Identification of pathologic features associated with "ulcerative colitis-like" crohn's disease. World journal of gastroenterology : WJG **20**, 13139–45 (2014)

[4] Mortlock, S., Lord, A., Montgomery, G., Zakrzewski, M., Simms, L., Krishnaprasad, K., Hanigan, K., Doecke, J., Walsh, A., Lawrance, I., Bampton, P., Andrews, J., Mahy, G., Connor, S., Sparrow, M., Bell, S., Florin, T., Begun, J., Gearry, R., Radford-Smith, G.: An extremes of phenotype approach confirms significant genetic heterogeneity in patients with ulcerative colitis (2021)

[5] Sudhakar, P., Verstockt, B., Cremer, J., Verstockt, S., Sabino, J., Ferrante, M., Md, P.: Understanding the molecular drivers of disease heterogeneity in crohn's disease using multi-omic data integration and network analysis. Inflammatory Bowel Diseases **27** (2020)
