# Supplementary material for: Large sample size and nonlinear sparse models outline epistatic effects in inflammatory bowel disease
Source: Genome Biol. 2023 Oct 5;24:224. doi: 10.1186/s13059-023-03064-y (PMC10552306; doi:10.1186/s13059-023-03064-y)
Supplement: Supplementary file 14 — Additional file 14: Table S6. Model hyperparameters. [file 13059_2023_3064_MOESM14_ESM.pdf]

Additional file 14: Table S6: Model hyperparameters

Model	Hyperparameters	Tuning range
Logistic regression with L_1 penalty	$alpha = 0.01$	$[1e-6, 1e-4, 1e-2, 1, 1e2, 1e4]$
Logistic regression with L_2 penalty	$alpha = 1$	$[1e-6, 1e-4, 1e-2, 1, 1e2, 1e4]$
Random forest classifier	n_estimators = 1000	$[1000, 100]$
	max_depth = 1000	$[None, 1000, 100, 3]$
$\mathrm{NN}_{\mathrm{logreg}}$	weightdecay = 0.1	$[0, 1e-5, 1e-3, 1e-1, 1]$
	dropout = 0.0	$[0, 0.1, 0.4, 0.75]$
	epochs = 20	$[10-20-30-50-75-100]$
$\mathrm{NN}_{\mathrm{dense}}$	weightdecay = 0.001	$[0, 1e-5, 1e-3, 1e-1, 1]$
	dropout = 0.4	$[0, 0.1, 0.4, 0.75]$
	epochs = 30	$[10-20-30-50-75-100]$
$\mathrm{NN}_{\mathrm{biosparse}}$	weightdecay = 0.001	$[0, 1e-5, 1e-3, 1e-1, 1]$
	dropout = 0.4	$[0, 0.1, 0.4, 0.75]$
	epochs = 30	$[10-20-30-50-75-100]$
