# Supplementary material for: Large sample size and nonlinear sparse models outline epistatic effects in inflammatory bowel disease
Source: Genome Biol. 2023 Oct 5;24:224. doi: 10.1186/s13059-023-03064-y (PMC10552306; doi:10.1186/s13059-023-03064-y)
Supplement: Supplementary file 15 — Additional file 15: Table S7. Tenfold cross-validation performance of best linear model and neural net. [file 13059_2023_3064_MOESM15_ESM.pdf]

Additional file 15: Table S7: Tenfold cross-validation performance of best linear model and neural net

Model	ROC AUC*
Best additive model	0.735 (0.00206)
$NN_{biosparse}$	0.760 (0.00567)

* Performance given as mean (standard deviation) of test set ROC AUC from 10 different full tenfold cross-validation runs with the same fold splits for all models.
