# Supplementary material for: Large sample size and nonlinear sparse models outline epistatic effects in inflammatory bowel disease
Source: Genome Biol. 2023 Oct 5;24:224. doi: 10.1186/s13059-023-03064-y (PMC10552306; doi:10.1186/s13059-023-03064-y)
Supplement: Supplementary file 16 — Additional file 16: Figure S6. Details of individual cross-validation runs for Fig. 2 on the performance of different random subsets containing 10%, 20%, 40%, 60%, 80%, and 100% of the dataset. [file 13059_2023_3064_MOESM16_ESM.pdf]

Additional file 16: Fig. S6: Details of individual cross-validation runs of Fig. 2 on the performance of different random subsets containing 10%, 20%, 40%, 60%, 80%, and 100% of the dataset.

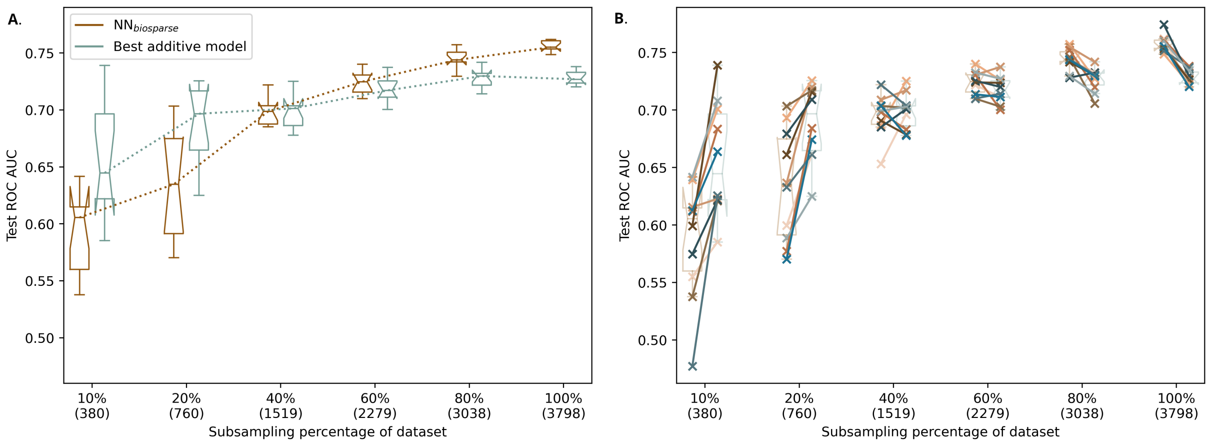

Figure 1: (A) Performance using different random subsets containing 10%, 20%, 40%, 60%, 80% and 100% of the dataset. Models shown are the best additive model (L_2 penalty) and $\mathrm{NN}_{\mathrm{biosparse}}$. Performances are measured using ten different threefold cross-validation runs, using identical splits for both models. (B) Same performance plot with lines connecting corresponding individual cross-validation runs with identical splits between the best linear model and the best neural network. Different colors represent different cross-validation run.
