# Supplementary material for: Large sample size and nonlinear sparse models outline epistatic effects in inflammatory bowel disease
Source: Genome Biol. 2023 Oct 5;24:224. doi: 10.1186/s13059-023-03064-y (PMC10552306; doi:10.1186/s13059-023-03064-y)
Supplement: Supplementary file 17 — Additional file 17. Review history. [file 13059_2023_3064_MOESM17_ESM.docx]

Review History

**First round of review**

**Reviewer 1**

**Were you able to assess all statistics in the manuscript, including the appropriateness of statistical tests used?**

Yes, and I have assessed the statistics in my report.

**Comments to author:**

The authors demonstrated the better performance of multiple neural network methods over additive models in the prediction of inflammatory bowel disease, which implied the non-linearity between IBD and WES data. The authors also showed the roles of sample size and the degree of sparsity, or more precisely, their ratio in outcome prediction. It is interesting to see the randomly selected genes outperforming KEGG annotated genes. By applying NN models, positive/negative epistasis in IBD was observed, while reciprocal sign epistasis was not. The work is supported by solid experiments and is worthy of publishment.

Major comments:

1. The outcome in this study is the IBD, a binary outcome, and a logistic lasso model was fitted to compare with the NNs. Thus rigorously speaking, the non-linearity that authors observed in this study was the log odds ratio of IBD risk. The question is, is it possible that other link functions in the GLM model rather than the logistic link work better?

2. There is one sentence describing the performance of WES PRS, but I didn't find technical detail of how PRS was constructed. I understand only a portion of the PRS SNPs are available in the WES data. Have the authors explored the lasso and NN models with the PRS SNPs as the only input features?

**Reviewer 2**

**Were you able to assess all statistics in the manuscript, including the appropriateness of statistical tests used?**

Yes, and I have assessed the statistics in my report.

**Comments to author:**

In this manuscript, the authors used the IBD WES dataset to demonstrate that NN-based nonlinear models outperform the traditional additive linear models when the sample size is large. They concluded that underdetermination is the major driver for the apparent optimality of additive modeling in clinical genetics. The implication is that more sophisticated nonlinear models are possible to build more accurate risk prediction model for large-scale genetic studies. Overall, the study is well designed, the manuscript is clearly written, the analyses are thorough, and the results seem to support their major conclusion. I have several comments the authors may consider to improve the statistical rigor:

1. What is the rationale of using three-fold CV? Are the results robust to other variants of CV?

2. It is important that the same training and test divisions are applied to all competing models. The authors should make clear about it. In Fig. 2, the authors should add connected lines between methods for the same division. By doing this, we know if the performance is consistent across runs.

3. The authors used a t-test to compare AUCs of different models based on the ten repetitions. However, different repetitions share a large trunk of training data, the AUCs are correlated. How to account for the correlation to derive a valid p-value? Please see Nadeau and Bengio, 2003, Machine Learning, 52, 239-281.

4. Suppl. Fig. 2 is very important since it proves that the improved prediction performance is not due to different input encoding. I think this is a better baseline than the logistic regression presented. I will suggest adding this to the main Fig. 2 and the table.

5. The inferior prediction performance of Random Forest can be due to the large number of input variables. Can the authors use the output of the shared gene module as the input to Random Forest? I think this is a fairer comparison.

**Authors Response**

**Point-by-point responses to the reviewers’ comments:**

Reviewer #1:

Comment 1: The outcome in this study is the IBD, a binary outcome, and a logistic lasso model was fitted to compare with the NNs. Thus rigorously speaking, the non-linearity that authors observed in this study was the log odds ratio of IBD risk. The question is, is it possible that other link functions in the GLM model rather than the logistic link work better?

*Response: We feel there might have been some misunderstanding about the meaning of the predictions scores the neural networks are producing. In Figure 3 we associate log odd ratios to the alleles for illustrative reasons, because we needed a metric that can be negative (i.e., indicating a protective effect of variants) to more clearly illustrate reciprocal sign epistasis. Besides that illustrative example, our neural network model produces individual-specific probability-like scores using a sigmoid function similar to the link function of the linear models used in the article. Using a different (monotonic) link function will not change the order of the predicted scores for the samples and thus will result in the same ROC AUC value.*

Comment 2: There is one sentence describing the performance of WES PRS, but I didn't find technical detail of how PRS was constructed. I understand only a portion of the PRS SNPs are available in the WES data. Have the authors explored the lasso and NN models with the PRS SNPs as the only input features?

*Response: We added a more detailed description on how the PRS was constructed in Supplementary Note S1, as well as the additional model analyses suggested by the reviewer on the selected 82 variants. The PRS, computed with the overlapping variants and GWAS-derived log odd ratios, reaches a ROC AUC of 0.563. None of the other models fitted on the 82 variants could improve on this, with a mean ROC AUC of 0.546 for tuned L1 and L2 penalized linear models, a mean ROC AUC of 0.553 for the random forest classifier and a mean ROC AUC of 0.536 for a neural network.*

Reviewer #2:

Comment 1: What is the rationale of using three-fold CV? Are the results robust to other variants of CV?

*Response: The choice to use three folds was motivated by concerns about the computation time required to produce the results, since for each model we tested we also performed 10 independent cross-validation runs to estimate the variance of the prediction performances. We nevertheless understand the concerns, and we have now added as Suppl. Table 7 the results of a tenfold cross-validation for the best linear model and the best neural network model, showing that for both the linear model and the neural network performance increases but the significant nonlinear advantage remains. In general, increasing the number of folds tends to (slightly) increase the prediction performance, since a larger proportion of the data is available as training set for each predicted sample.*

Comment 2: It is important that the same training and test divisions are applied to all competing models. The authors should make clear about it. In Fig. 2, the authors should add connected lines between methods for the same division. By doing this, we know if the performance is consistent across runs.

*Response: As the reviewer mentioned, by using random splits for each cross-validation run, the exact similarity of the splits used for the different models we compare is not guaranteed. To address the concern, we re-ran all analyses of Table 1 and Fig. 2, 4 and 5 using the exact same splits. We can see that the results are very similar and the order between the linear and nonlinear methods performances remain the same.*

*We did not re-run the ensemble models computation shown in Table 2, since 1) Table 1 and Fig. 2, 4 and 5 show that the same patterns occur regardless of the cross-validation procedure used, and 2) the extensive computational effort needed to train our ensemble NN models.*

*To address the last remark, we added in Supplementary (Suppl. Fig. 6) a figure showing the connections between the same folds between the neural net and the linear model in a cross-validation setting using the same splits.*

Comment 3: The authors used a t-test to compare AUCs of different models based on the ten repetitions. However, different repetitions share a large trunk of training data, the AUCs are correlated. How to account for the correlation to derive a valid p-value? Please see Nadeau and Bengio, 2003, Machine Learning, 52, 239-281.

*Response: We fully agree and have recomputed p-values according to the approach from the suggested article. As expected, p-values became bigger but remained significant.*

Comment 4: Suppl. Fig. 2 is very important since it proves that the improved prediction performance is not due to different input encoding. I think this is a better baseline than the logistic regression presented. I will suggest adding this to the main Fig. 2 and the table.

*Response: Following the suggestion, we added the performance of the linear neural network to Fig. 2 and Table 1.*

Comment 5: The inferior prediction performance of Random Forest can be due to the large number of input variables. Can the authors use the output of the shared gene module as the input to Random Forest? I think this is a fairer comparison.

*Response: The performances of the suggested extra random forest analyses are added to the Supplementary (Suppl. Table 3). Somewhat unexpectedly, we did not see an increase over the performance of the random forest using the individual variants.*

**Second round of review**

**Reviewer 1**

All my comments have been addressed.

**Reviewer 2**

The authors have addressed my concerns satisfactorily.
